# Are rapid diagnostic tests more accurate in diagnosis of malaria compared to microscopy?

**DOI:** 10.1186/1475-2875-9-S2-P5

**Published:** 2010-10-20

**Authors:** Vincent Batwala, Pascal Magnussen, Fred Nuwaha

**Affiliations:** 1 Department of Community Health, Mbarara University of Science & Technology, P. O. Box 1410, Mbarara, Uganda; 2 Centre for Health Research and Development, Faculty of Life Sciences, Copenhagen University, Thorvaldsensvej 57, DK1871 Frederiksberg C, Denmark; 3 Disease Control and Environmental Health, Makerere University School of Public Health, P. O. Box 7072, Kampala, Uganda

## Background

Prompt, accurate diagnosis and treatment with artemisinin combination therapy remains vital to current malaria control. Blood film microscopy the current standard test for diagnosis of malaria has several limitations that necessitate field evaluation of alternative diagnostic methods especially in low income countries of sub-Saharan Africa where malaria is endemic.

## Methods

We compared the accuracy of axillary temperature, health centre microscopy, expert microscopy and a HRP2-based rapid diagnostic test (Paracheck) in predicting malaria infection using polymerase chain reaction (PCR) as the gold standard. Three hundred patients with a clinical suspicion of malaria based on fever and or history of fever from a low and high transmission setting in Uganda were consecutively enrolled and provided blood samples for all tests. The endpoints were: sensitivity, specificity, positive predictive value (PPV) and negative predictive value (NPV). This study is registered with Clinicaltrials.gov, NCT00565071.

## Results

Of the 300 patients, 88(29.3%) had fever, 56(18.7%) were positive by health centre microscopy, 47(15.7%) by expert microscopy, 110(36.7%) by Paracheck and 89(29.7%) by PCR. The overall sensitivity >90% was only shown by Paracheck 91.0% [95%CI: 83.1-96.0]. The sensitivity of expert microscopy was 46%, similar to health centre microscopy. The superior sensitivity of Paracheck compared to microscopy was maintained when data was stratified for transmission intensity as well as by age. The overall specificity rates were: Paracheck 86.3% [95%CI: 80.9-90.6], health centre microscopy 93.4% [95%CI: 89.1-96.3] and expert microscopy 97.2% [95%CI: 93.9-98.9]. The NPV >90% was shown by Paracheck 95.8% [95%CI: 91.9-98.2]. The overall PPV was <88% for all methods.

## Conclusion

High sensitivity of malaria diagnosis is essential because the infection produces an acute illness in vulnerable populations that can rapidly progress to death. The HRP2-based rapid diagnostic test has shown superior sensitivity compared to microscopy and may be more suitable for screening of malaria infection.

